# The Immune Checkpoint Receptor CD96: A Local and Systemic Immune Modulator in Oral Cancer?

**DOI:** 10.3390/cancers15072126

**Published:** 2023-04-02

**Authors:** Leah Trumet, Manuel Weber, Alina Hahn, Lina Kunater, Carol Geppert, Jacek Glajzer, Ann-Kristin Struckmeier, Tobias Möst, Rainer Lutz, Marco Kesting, Jutta Ries

**Affiliations:** 1 Department of Oral and Cranio-Maxillofacial Surgery, Friedrich-Alexander-Universität Erlangen-Nürnberg (FAU), 91054 Erlangen, Germany; manuel.weber@uk-erlangen.de (M.W.); jutta.ries@uk-erlangen.de (J.R.); 2 Department of Operative Dentistry and Periodontology, Friedrich-Alexander-Universität Erlangen-Nürnberg (FAU), 91054 Erlangen, Germany; 3 Deutsches Zentrum Immuntherapie (DZI), Comprehensive Cancer Center Erlangen-EMN (CCC ER-EMN), Friedrich-Alexander-Universität Erlangen-Nürnberg (FAU), 91054 Erlangen, Germany; 4 Institute of Pathology, Friedrich-Alexander-Universität Erlangen-Nürnberg (FAU), 91054 Erlangen, Germany

**Keywords:** immune checkpoints, immunotherapy, OSCC, HNSCC, PD-1, PD-L1

## Abstract

**Simple Summary:**

As immune checkpoint inhibitor (ICI) therapy against PD1 is only efficient in a small proportion of OSCC patients, identification of further checkpoints might improve therapy response by enabling combination ICI treatment. The aim of this study was to analyze the gene- and protein-expression of the checkpoint CD96 in tissue and peripheral blood of OSCC patients compared to healthy controls, while also checking for associations to histomorphological parameters. Patients and controls were analyzed by real-time quantitative polymerase chain reaction and by immunohistochemistry. CD96 expression in tumor tissue and peripheral blood of OSCC patients is differentially regulated. Tumor tissue showed a significant upregulation of CD96 expression. mRNA and protein expression correlated significantly. In peripheral blood of OSCC patients a significant downregulation of CD96 was observable. CD96 expression correlated with other immune checkpoints. CD96 might be a relevant immune checkpoint and needs further investigation especially in the context of immunotherapy.

**Abstract:**

**Background**: As immunotherapy of oral squamous cell carcinomas (OSCCs), using PD1 inhibitors, is only efficient in a small proportion of patients, additional immune checkpoints need to be identified as potential therapeutic targets. There is evidence that a blockade of CD96 might positively affect the anti-tumor immune response. The aim of this study was to analyze the gene and protein expression of CD96 in the tissue and peripheral blood of OSCC patients compared to healthy controls, while also checking for potential associations with a differential expression to the histomorphological parameters. In addition, possible correlations with the expression of PD1 and PD-L1 as well as the macrophage markers CD68 and CD163 should be tested to obtain further insights into the potential effectiveness of combined checkpoint blockage. **Material and Methods:** For real-time quantitative polymerase chain reaction (RT-qPCR), a total of 183 blood and tissue samples, divided into a patient and a control group, were included. Additionally, 141 tissue samples were examined by immunohistochemistry (IHC). The relative expression differences between the groups were calculated using statistical tests including the Mann–Whitney U test and AUC method. The Chi-square test was used to determine whether CD96 overexpression in individual samples is associated with malignancy. Correlation analysis was performed using the Spearman correlation test. **Results:** There was a significant CD96 mRNA and protein overexpression in the OSCC group compared to the controls (*p* = 0.001). In contrast, CD96 mRNA expression in the peripheral blood of the OSCC patients was significantly lower compared to the control group (*p* = 0.007). In the Chi-square test, the OSCC tissue samples showed a highly significant upregulation of CD96 mRNA expression (*p* < 0.001) and protein expression (*p* = 0.005) compared to the healthy mucosa. CD96 mRNA and protein expression correlated significantly (*p* = 0.005). In addition, there was a significant positive correlation of CD96 expression with PD1 (*p* ≤ 0.001), PD-L1 (*p* ≤ 0.001), and CD163 (*p* = 0.006) at the mRNA level. **Conclusions:** CD96 expression in the tumor tissue and peripheral blood of OSCC patients is differentially regulated and appears to be a relevant immune checkpoint.

## 1. Introduction

The introduction of immunotherapy (IT) using immune checkpoint inhibitors (ICIs) has significantly expanded treatment options for oral squamous cell carcinomas (OSCCs) [1]. However, despite the success of ICI therapy, around 66–85% of patients do not respond or do not show significant improvements in tumor treatment [2].

One explanation might be the immune cell composition in these tumors. Tumors with a lack of T-cell infiltration are considered as immunological “cold” tumors. In contrast to “hot” tumors with high immune infiltration, ICI therapy can be less effective in “cold” tumors [3]. A recent analysis of OSCCs could identify 46% of cases as “hot” and 54% as immunological “cold” by using a multi-marker panel [4]. The immunological “hot” tumors also showed a significantly better survival when treated without the use of immunotherapy [4].

One strategy to improve ICI response is to advance its use from the palliative setting to earlier disease stages, leading to neoadjuvant treatment protocols prior to surgery [5,6]. Another attempt is the combination of multiple ICIs to increase response rates [7]. Currently, inhibition of the immune checkpoint PD1 is the only approved ICI treatment in OSCCs [8]. PD1 is an inhibitory receptor of T cells that can be activated by the PD-L1 and PD-L2 ligands that are expressed by tumor cells but also by immune cells such as antigen-presenting cells (APCs) including macrophages [9,10,11].

One relevant mechanism of tumor immune escape is the downregulation of MHC class I molecules (MHC I) on the surface of tumor cells, which are essential for the recognition of tumor antigens by T cells and, therefore, T cell-dependent tumor clearance [12]. However, natural killer cells (NK cells) can detect and kill MHC I negative cells [12]. Therefore, NK cells are important cells in the anti-cancer immune response. Analog to T cells, their function can be regulated by activating or inhibiting immune checkpoints [12].

The identification of further immune checkpoint pathways in addition to PD1 in OSCCs could smooth the road for future, more advanced treatment strategies. Using a nano-string assay on the mRNA level, a set of potentially relevant checkpoint pathways in OSCCs were identified [13]. The CD96 pathway is one of the identified candidates [13].

The function of the CD96 pathway is currently insufficiently understood [14,15]. The CD96 receptor, also known as TACTILE, is expressed on T cells and NK cells. It interacts with the CD155 ligand which is expressed on APCs including macrophages and also on tumor cells [12,16,17].

CD96 is involved in a relatively complex signaling pathway as CD96 and the inhibitory TIGIT receptor compete with the co-stimulatory CD226 receptor for binding to the CD155 ligand [17]. Furthermore, there is a correlation between CD226 and CD96 on activated human T cells and intratumoral NK cells [17]. Database analyses including the Oncomine and TCGA gene expression database show a high expression of CD96 in brain cancer, breast cancer, head-and-neck squamous cell carcinoma (HNSCC), and other malignant diseases but a low expression in lung cancer, rectal cancer, and others [15,18].

CD96 mRNA expression in human tissue samples of different cancer types showed a high correlation with T-cell markers and also with the PD1 checkpoint receptor [19]. In patients with hepatocellular carcinomas, a high expression of CD96 and CD155 was associated with decreased survival [20]. In cervical cancer, CD96 expression on the CD8^+^ T cells of patients not responding to anti-PD1 immunotherapy was increased [21]. In the peripheral blood of pancreatic cancer patients, a significant decrease in CD96^+^ NK cells was shown in the flow cytometry [16]. In mouse models, CD96 blocking significantly increased the effect of anti-PD1 ICIs and improved the function of the CD8^+^ T cells [21].

In an immunohistochemical (IHC) analysis in elderly HNSCC patients treated with radio- or radio-chemotherapy, there was no association between CD96 expression and survival [22]. Data analysis using the Cancer Genome Atlas in 49 HPV^+^ HNSCC patients revealed better survival in cases of increased CD96 expression. In contrast, there was no association with survival in HPV^-^ cases [14].

The current study aimed to analyze the expression of CD96 in the tumor tissue and peripheral blood of OSCC patients compared to the mucosa and blood samples of healthy volunteers on the mRNA and protein level. In addition, possible associations between CD96 expression, clinical, demographic, and histomorphologic parameters, including TNM stage and grading, should be identified. Moreover, the CD96 mRNA expression should be correlated with the protein expression in tumor and mucosa specimens, as well as the expression of the immune checkpoints PD1 and PD-L1 and the macrophage markers CD68 and CD163.

## 2. Material and Methods

### 2.1. Patients Collective

For this retrospective study, tissue specimens (tumor and healthy mucosa), as well as peripheral whole blood samples from patients with oral squamous cell carcinomas (OSCCs) and healthy (regarding malignancy and infection) control persons, were examined. The samples were taken from individuals aged between 18 and 89 years who were treated at the FAU Erlangen-Nuremberg between 2010 and 2021. Inclusion in the study was performed with the full consent of the patients and control group and with the approval of the ethics committee (application number 3962). The collective was divided into two groups. The OSCC patients group included both blood and tissue samples from individuals diagnosed for the first time with oral cancer and the control group included normal oral mucosa specimens as well as blood samples from healthy volunteers. Of the OSCC patients, 3 received neoadjuvant treatment with Pembrolizumab 200 mg approximately 10 days prior to surgery.

OSCC patients were classified according to the size of the tumor (T1–T4) (in addition, smaller-sized tumors (T1 + T2) and larger ones (T3 + T4) were grouped), negative or positive lymph node status (N0 and N+) to indicate the absence (N0) or presence (N+) of lymphatic metastases, and their L-status (subdivided into the absence (L0) or invasion into lymphatic vessels (L1) and their grade of differentiation (well differentiated (G1), moderately (G2), poorly differentiated (G3)). Another parameter for classification was the status of perineural invasion (Pn1 = invasion and Pn0 = no perineural invasion).

The number of samples and the demographic and histomorphological parameters of both groups that were analyzed by RT-qPCR and IHC are summarized in Table 1(a,b).

Due to the limited amount of material and a slightly different time period, not all samples were analyzed by both methods, so Table 1(a) shows the exact parameters of the 183 cases, 98 OSCC patients, and 85 normal mucosa controls measured by RT-qPCR, and Table 1(b) shows the characteristics of the 141 samples, 90 OSCC patients, and 51 healthy volunteers who were analyzed by IHC. In 78 cases, including OSCC- and control-group, both IHC of tissue and RT-qPCR analysis of blood was carried out. In 68 cases, IHC analysis of tissue, and RT-qPCR of blood and tissue could be performed.

### 2.2. Processing of Tissue and Blood

The tissue specimens of healthy volunteers (NOM) were collected during minor oral surgical procedures, such as the removal of wisdom teeth, which did not increase the size of the procedure or harm the patient. All malignant tissue samples were collected in the primary OSCC surgery. The harvested specimens were divided into two pieces. One of these was fixed in formalin and examined by the Institute of Pathology, FAU Erlangen-Nuremberg for TNM classification (OSCC) and absence of inflammation (NOM). Additionally, these formalin-fixed and paraffin-embedded tissue samples were used for IHC analysis. The other part of the sample was transferred into RNAlater^®^ (Qiagen, Hilden, Germany). After transfer to RNAlater^®^, samples were fixed by incubation at 4 °C for a minimum of 24 h and were then stored at −80 °C until mRNA isolation.

From OSCC patients and healthy controls, 2.5 mL whole peripheral blood was collected in a PAXgene Blood RNA Tube (PreAnalytiX GmbH, Hombrechtikon, Switzerland). Blood samples were obtained from healthy volunteers before surgery as well as from OSCC patients directly before tumor surgery. The samples were carefully inverted 8–10 times, incubated at room temperature for 2 hours, and stored at −20 °C for 24 h. Storage up to RNA isolation was carried out at −80 °C.

### 2.3. Analysis of CD96 Expression by Quantitative Real-Time Reverse Transcriptase Polymerase Chain Reaction (RT-qPCR)

After tissue disruption using a Precellys^®^ (Bertin Instruments Company, Montigny-le-Bretonneux, France), total RNA was isolated using the Qiagen “miRNeasy mini-Kit” (Qiagen Company, Hilden, Germany) according to the manufacturer’s instructions.

The quality and quantity of the RNA samples were determined using the Nano-Drop 3.3 ND1000 spectrophotometer and the corresponding ND-1000 software (Thermo Fisher Scientific Company in Waltham, MA, USA, V3.8).

Reverse transcription of total RNA into cDNA was performed using the Transcriptor High-Fidelity cDNA Synthesis Kit according to the manufacturer’s recommendations (Roche, Mannheim, Germany).

For further analysis of CD96, PD1, PD-L1, CD68, and CD163 expression in the specimen, gene-specific primers (Table 2) and PowerSybrGreen Mastermix (Life Technologies, Darmstadt, Germany) were used. For CD96 quantification, two transcript variants (CD96_1 and CD96_3) were used. The amplification of transcript variant 1 (CD96_1) encodes the longer isoform compared to CD96_3.

The primer length varies from 20 bp to 23 bp, and the amplicon length from 91 bp to 152 bp. The annealing temperature for the CD96, PD1, PD-L1, CD68, and CD163 primer pairs was 60 °C. The number of cycles performed by PCR amounted to 40. The study’s data were collected and analyzed using the ABI Prism 7300 from Applied Biosystems (ThermoFisher Scientific Inc., Waltham, MA, USA). Primer sequences, cycling conditions, and genes used for primer design (clearly defined by their accession numbers) are summarized in Table 2.

The data were normalized by the ΔCT method using GAPDH as an internal control. These data were used for statistical analysis. The relative quantification of differences in gene expression between the two groups was based on the ∆∆CT-method (RQ = fold change (FC) = 2^−ΔΔCT^).

### 2.4. Detection and Quantitative Immunohistochemical Analysis of CD96 Expression by Immunohistochemistry (IHC)

From the tissue samples fixed in formalin and embedded in paraffin, 2 µm sections were prepared with the Rotary Microtome (RM2165, Leica Biosystems, Wetzlar, Deutschland), fixed in the 57 °C heat cabinet on glass slides (Superfrost Plus Gold Adhesion Microscope Slides, White Tab, Epredia, Portsmouth, NH, USA) and subsequently examined histopathologically.

A cut of all samples was additionally stained with H&E and analyzed using a microscope to ensure that a relevant amount (<70%) of malignant tissue content was present in the tissue specimens of the OSCC group and only healthy, inflammation-free, or almost inflammation-free oral mucosa was present in the control group.

After pretreatment of the slides in a water bath at 100 °C for 30 min with antigen retrieval buffer (citrate buffer, pH 6.0, Medac MAD-004071R/D) followed by cooling down in the citrate buffer at room temperature for 30 min and afterward for 5 min in the wash buffer (pH 7.6, DAKO, S3006), the samples were analyzed for CD96 expression applying the anti-CD96 antibody (Abcam, ab264416, GR13312304-3, dilution 1:200) according to the manufacturer‘s recommendations. Human tonsils served as the positive control and human oral mucosa with antibody diluent served as the negative control.

The slides were scanned and digitalized using the method of “whole slide imaging” with 40× magnification and the Panoramic 250 Flash III Scanner in the Department of Pathology.

**Figure 1:**
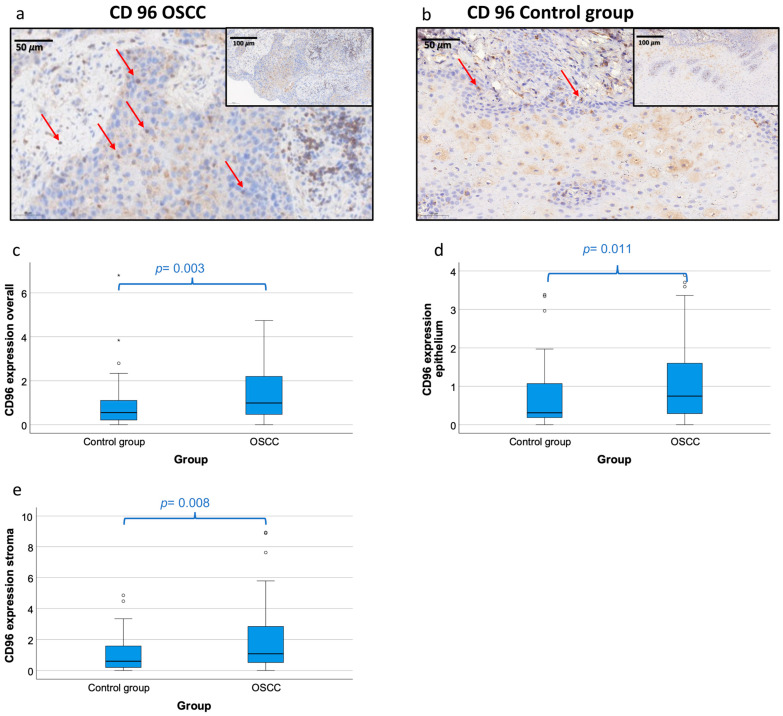
CD96 protein expression in OSCC tumor tissue and healthy control mucosa. (**a**,**b**) Representative micrographs showing the typical expression pattern of CD96 in OSCC tissue (**a**) and healthy control mucosa (**b**). All micrographs are given in high-power magnification (30× magnification) and panoramic magnification (15× magnification). CD96 staining shows a cytoplasmatic expression pattern with accentuation of the plasma membrane. CD96 expressing cells are indicated with arrows. (**c**–**e**) Box plots of the median CD96 protein expression rates in tumor tissue of OSCC patients (OSCC) and oral mucosa of healthy volunteers (control group). The median labelling indices (positive cells vs. all cells) of CD96-expressing cells in the complete analyzed tissue (CD96 expression overall, (**c**)), the epithelial tumor and mucosa compartment (CD96 expression epithelium, (**d**)), and in the stroma tissue (CD96 expression stroma, (**e**)) provided by immunohistochemistry are given. Higher labelling indices indicate higher CD96 protein expression. The median, the interquartile range, and the standard deviation are provided. Statistical analyses were carried out using the Mann–Whitney U test. ° outliners.

A total of 3 equally sized rectangular (0.5 mm^2^) image fields (region of interest, ROIs) were created for each sample by the Case Viewer 2.3 software (3DHISTECH^®^, Budapest, Hungary). Subsequently, these ROIs were subdivided into epithelium and stroma and a TIF file was exported for each field of view for the entire ROI, the epithelial part, and the stromal part and then analyzed using QuPath 0.4.1 [21]. An automatic analysis of the overall cell count in all ROIs was performed. Subsequently, the CD96-expressing cells were manually labeled and counted in a standardized manner in the Case Viewer to determine the (labelling index, (LI)). The LI was determined by the number of positive cells divided by the overall cell count in each ROI. Then, the average LI of all three ROIs per sample was used for statistical analysis. Only cells with a clear positive CD96 staining were counted (Figure 1a,b).

## 3. Statistical Analysis

In RT-qPCR analysis, the relative CD96 gene expression between two groups, represented as the fold change (FC), was calculated according to the ∆∆CT method. A value greater than 2 implies a relevant increase. The FC in the immunohistological staining corresponded to the ratio of the mean LI of the groups (∆LI1/∆LI2).

For the statistical analysis, based on the data collection from the RT-qPCR (ΔCT) and the IHC (ΔLI) values, the statistical software package SPSS 23 (SPSS Inc., Chicago, IL, USA) was used.

First, the data were analyzed in an exploratory data analysis. Nonparametric tests such as Mann–Whitney U were used to determine whether expression levels significantly differed between groups. A *p*-value ≤ 0.05 was considered statistically significant, and a *p*-value ≤ 0.001 was considered highly significant.

Furthermore, the expression profile of CD96 was used to create receiver operator characteristic (ROC) curves. This method displays the discriminatory accuracy of the marker for distinguishing between two groups. It is a plot of the sensitivity (true-positive rate) vs. 1-specificity (false-positive rate) over all possible threshold values of the marker. The area under the ROC curve (AUC) value defines the usefulness of a marker to separate the two different groups (OSCC patients/controls). An AUC value of 0.5 means that no differentiation by the marker is possible. The closer the value approaches 1 or 0, the better its suitability is for classification. A good classifier predicts a positive point that has a high probability to, indeed, be positive (specificity). On the other hand, one wants a high sensitivity, i.e., as many samples as possible that are positive should be detected as true-positive. Thus, the classifier was chosen in such a way that the highest possible sensitivity is accompanied by the highest possible specificity. This value is defined as the highest Yourdon (Y)-index.

The Y-index was used to calculate a “cut-off-point” (COP) for CD96 expression which can classify a sample as negative or positive regarding overexpression. This classification can then be used to determine the correlation between malignancy and positivity. That is, whether a positive classification is also associated with an OSCC disease. Thus, the significance, as well as sensitivity and specificity of the investigated marker, can be calculated [23]. Based on these COP values, the two groups were divided into two subgroups which showed an expression rate above and below the COP. Afterward, the statistical association between increased or decreased CD96 expression with the presence of malignancy was calculated using the Chi-square test (χ^2^ test).

A correlation analysis of the mRNA CD96 expression in the tissue samples obtained from RT-qPCR with the protein expression derived from IHC was performed. In addition, the CD96 mRNA expression levels determined were correlated with the mRNA expression of CD68 and CD163 as well as the previously published data of PD-L1 [9] and PD1 [10]. Correlation analysis was performed by Spearman’s correlation test. Spearman correlation values and two-sided adjusted *p*-values are provided.

## 4. Results

### 4.1. Demographic and Clinico-Histopathological Characteristics of the Study Collective

The demographic, clinical, and histomorphological parameters of the study collective are summarized in Table 1. Table 1(a) shows the collective subjected to RT-qPCR analysis and Table 1(b) the collective for immunohistochemistry (IHC). The mean age of the healthy control group was significantly lower than in the OSCC group (*p* < 0.001). None of the healthy volunteers had relevant oral mucosal changes, such as inflammation, hyperplasia, or dysplasia.

Out of the 98 OSCC patients in the RT-qPCR collective, 61 had small (T1/T2: 62.3%) and 32 had large tumors (T3/T4: 32.7%). In 53 of the cases, the lymph nodes were not affected (N0: 54.1%). In total, 9 out of the OSCC tissues were mildly (G1: 9.2%), 46 moderately (G2: 47%), and 37 severally (G3: 37.7%) dedifferentiated. A total of 70 cases did not show lymph vessel infiltration (L0: 71.4%) and 18 cases had lymphatic infiltration (L1: 18.4%). A total of 59 cases were Pn0 (60.2%) and 28 (28.6%) were Pn1 (Table 1(a)).

The three patients receiving preoperative Pembrolizumab application showed a tendency for increased CD96 expression in the tissue samples. Statistical tests were not possible due to the small number of cases. Exclusion of the three cases did not change the remaining statistical results.

The collective for IHC analysis shows a similar composition of histomorphological characteristics with a slightly higher proportion of T3/T4 cases (Table 1(b)).

### 4.2. Immunohistochemical Staining Characteristic of CD96

In the IHC analysis, CD96 showed cytoplasmatic staining with an accentuation of the cell membrane. The tumor stroma showed significantly higher CD96 labeling indices compared to the epithelial compartment (p_OSCC_ = 0.047). Most CD96-expressing cells had immune cell-like morphology (Figure 1a,b). In the epithelial compartment of the tumor and stroma samples, some cells with epithelial cell/tumor cell morphology also showed discrete CD96 expression. In the control samples, there was no significant difference in CD96 expression between the epithelial and the subepithelial layer (p_healthy controls_ = 0.286).

### 4.3. Comparison of CD96 Expression in Peripheral Blood between OSCC Patients and Healthy Controls

**Figure 2:**
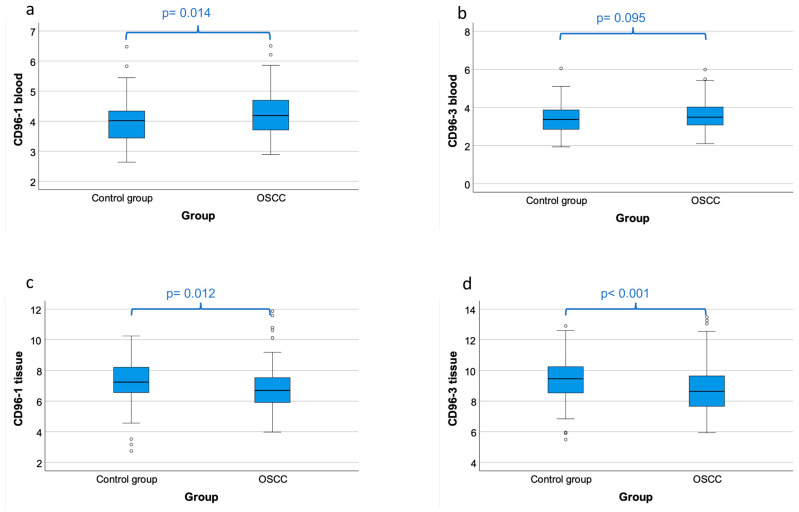
CD96 mRNA expression in peripheral blood and tumor tissue of OSCC patients and controls. Box plots showing the median CD96 mRNA expression rates in peripheral whole blood samples of OSCC patients (OSCC) and healthy volunteers (control group) (**a**,**b**) as well as tumor tissue of OSCC patients and healthy oral mucosa of volunteers (**c**,**d**). The median ΔCT values of CD96 transcript variant 1 (**a**,**c**) and transcript variant 3 (**b**,**d**) expression levels derived from RT-qPCR are given. Lower ΔCT values indicate higher CD96 mRNA expression. The median, the interquartile range, and the standard deviation are provided. Statistical analyses were carried out using the Mann–Whitney U test. ° outliners.

**Figure 3:**
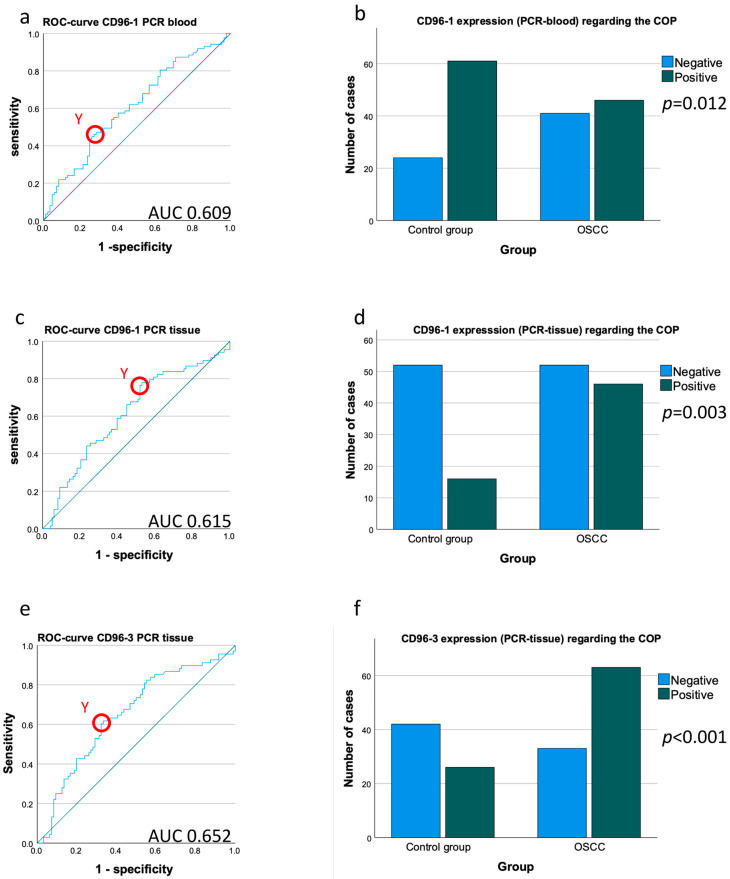
Determination of a cut-off point and allocation of individual cases to a group (controls vs. OSCC patients) based on CD96 mRNA expression. (**a**,**c**,**e**) ROC curves for CD96 mRNA expression based on the ∆CT data. The diagrams are a plot of the sensitivity (true-positive rate) vs. 1-specificity (false-positive rate) over all possible ∆CT values. The circles show the points of the highest Youden (Y) indices which are associated with the COP (OSCC patients vs. controls). The AUC value is indicated. ROC: receiver operating characteristic, COP: cut-off point, AUC: area under the curve. (**b**,**d**,**f**) Division of the test and control group (group OSCC patients and group controls) into positive and negative subgroups based on the ascertained COPs of CD96 expressed as ∆CT values. Using the χ^2^ test, the specimens were positively (malignant) judged if the values lay above the COP (decreased expression) in blood samples (**a**) and below the COP (increased expression) in tissue samples (**d**,**f**). Decreased CD96 expression levels in the peripheral blood (**b**) and increased CD96 expression levels in the tissue of OSCC patients (**d**,**f**) compared to the healthy oral mucosa of volunteers were significant. Therefore, the COP may be a parameter allowing the allocation of a blood or tissue sample to a group and an indication of malignancy. (**a**) ROC curve for CD96 transcript variant 1 in peripheral blood samples. (**b**) χ^2^ test for CD96 transcript variant 1 in peripheral blood samples. (**c**) ROC curve for CD96 transcript variant 1 in tumor tissue and mucosa samples. (**d**) χ^2^ test for CD96 transcript variant 1 in tumor tissue and mucosa samples. (**e**) ROC curve for CD96 transcript variant 3 in tumor tissue and mucosa samples. (**f**) χ^2^ test for CD96 transcript variant 3 in tumor tissue and mucosa samples.

Expression of the CD96 transcript variant 1 (CD96_1) in the peripheral blood samples of the OSCC patients was significantly lower compared to the healthy controls (mean ∆CT patients 4.24, mean ∆CT controls 3.99; *p* = 0.014) (Table 3, Figure 2a). The downregulation of CD96 expression in the blood of the OSCC patients was significant and amounted to a negative 0.84-fold change in the expression rates (Table 3). The significance of the CD96_1 downregulation was confirmed by the AUC value (0.609) (Table 3, Figure 3a). The highest Youden index was 0.189 for CD96_1 (Figure 3a). The optimal threshold ∆CT value (COP) for distinguishing the OSCC patients from the healthy controls was 4.29 (Table 3). Using the determined COP, the two groups (patients and controls) were separated into positive and negative cases (Figure 3b). Therefore, it should be confirmed that CD96 expression allows the detection of malignancy in a certain blood sample. The statistical evaluation by the Chi-square test revealed that the decreased expression rate of CD96_1 was statistically associated with malignancy (*p* = 0.012). The results are summarized in Table 3 and illustrated in Figure 3b.

Expression of the CD96 transcript variant 3 (CD96_3) was not significantly downregulated in the OSCC patients compared to the control group (Table 2, Figure 2b).

### 4.4. Comparison of CD96 Expression in Tissue Samples between OSCC Patients and Healthy Controls

The CD96 expressions of transcript variant 1 (CD96_1) and transcript variant 3 (CD96_3) in the OSCC tissue samples measured by RT-qPCR were significantly higher (lower ∆CT-value) compared to the healthy controls (mean ∆CT_CD96_1_ patients 6.84; mean ∆CT_CD96_1_ controls 7.20; *p* = 0.012; mean ∆CT_CD96_3_ patients 8.78, mean ∆CT_CD96_3_ controls 9.38; *p* < 0.001) (Table 3, Figure 2c,d).

The upregulation of CD96 in the OSCC tissue samples was significant for CD96_1 and highly significant for CD96_3 and amounted to a 1.28 _CD96_1_ and a 1.52 _CD96_3_-fold change in the expression rates (Table 3). Furthermore, the significance of the CD96_1 upregulation was confirmed by the AUC values (AUC_CD96_1_ = 0.615, AUC_CD96_3_ = 0.652) (Table 3, Figure 3c,e). The highest Youden index was 0.248 for CD96_1 (Figure 3c) and 0.285 for CD96_3 (Figure 3e). The most appropriate threshold ∆CT value (COP) for differentiating the OSCC patients from the healthy controls was 6.52 for CD96_1 and 9.27 for CD96_3 (Table 3). Based on the determined COP, the control and patient groups were divided into positive and negative cases regarding the CD96 expression above and below the COP (Figure 3d,f).

Statistical analysis using the Chi-square test proved that a higher expression level of CD96 in an individual tissue sample is highly associated with malignancy (p_CD96_1_ = 0.003, p_CD96_3_ < 0.001; Figure 3d,f). The data are presented in Table 3 and Figure 3d,f.

**Figure 4:**
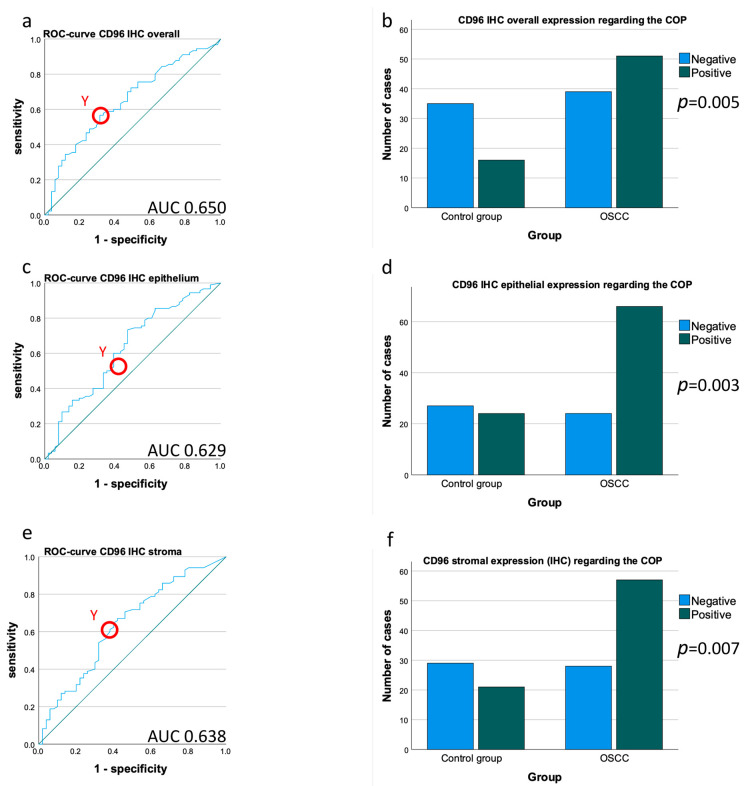
Determination of a cut-off point and allocation of individual cases to a group (controls vs. OSCC patients) based on CD96 protein expression. (**a**,**c**,**e**) ROC curves for CD96 mRNA protein expression based on LI determined by immunohistochemistry (IHC). The diagrams are a plot of the sensitivity (true-positive rate) vs. 1-specificity (false-positive rate) over all possible labeling indices. The circles show the points of the highest Youden (Y) indices which are associated with the COP (patients vs. controls). The AUC value is indicated. ROC: receiver operating characteristic, COP: cut-off point, AUC: area under the curve. (**b**,**d**,**f**) Division of the test and control group (group OSCC patients and group controls) into positive and negative subgroups based on the ascertained COPs of CD96 expressed as labeling index values. Using the χ^2^ test, the specimens were positively (malignant) judged if the values lay above the COP. Increased CD96 expression levels in the tissue of OSCC patients (**d**,**f**) compared to the healthy oral mucosa of volunteers were significant. Therefore, the COP may be a parameter allowing the allocation of the tissue sample to a group and an indication of malignancy. (**a**) ROC curve for CD96 in the complete tissue area (epithelial + stromal). (**b**) χ^2^ test for CD96 protein expression in the complete tissue area (epithelial + stromal). (**c**) ROC curve for CD96 in the epithelial tissue compartment. (**d**) χ^2^ test for CD96 protein expression in the epithelial tissue compartment. (**e**) OC curve for CD96 in the stromal tissue compartment. (**f**) χ^2^ test for CD96 protein expression in the stromal tissue compartment.

In addition to the described mRNA assessments, an analysis of the IHC-determined protein expression rate (labeling index, LI) of the CD96 tissues was performed. CD96 protein expression in the OSCC group was significantly higher compared to the healthy controls (mean LI_CD96_overall_ OSCC 1.39, mean LI_CD96_overall_ controls 0.91; *p* = 0.003) (Table 3, Figure 1a–c). The AUC value (0.650) confirms the upregulation of CD96 protein expression in the OSCC collective (Table 3, Figure 4a). The highest Youden index was 0.253 for CD96 (Figure 4a).

The most appropriate threshold (COP), which allows the separation of the controls and patients, was an IL = 0.905 (Table 3). The Chi-square test analysis showed that an increased labeling index of CD96 (above the COP) in the tissue samples was associated with the presence of malignancy (*p* = 0.005; Figure 4b).

Moreover, separate expression analysis of CD96 in the tumor and healthy mucosa epithelium and the tumor and subepithelial stroma was performed. The CD96 expression in the epithelium and stroma of the healthy mucosa was lower compared to the OSCCs (mean LI_CD96_epithelium_ OSCC 1.09, mean LI_CD96_epithelium_ controls 0.85; *p* = 0.01; mean LI_CD96_stroma_ patients 2.11, mean LI_CD96_stroma_ controls 1.52; *p* = 0.008) (Table 3, Figure 1d,e). This shows that even when the tissue is subdivided into epithelium and stroma, the results are concomitant, which is supported by the AUC values (AUC_CD96_overall_ = 0.652; AUC_CD96_epithelium_ = 0.629, AUC_CD96_stroma_ = 0.638) (Figure 4a,c,e).

Other statistical tests also show confirmation of the results found in the overall tissue; for example, the Chi-square test (p_CD96_epithelium_ = 0.003, p_CD96_stroma_ = 0.007) showed confirmation of a higher expression in the OSCCs compared to the healthy oral mucosa (Figure 4d,f).

The detailed results are presented in Table 3, Figure 2 and Figure 4.

### 4.5. Association CD96 Expression Patterns with Histomorphological Parameters

It was tested if histomorphological parameters, including T-, N-, L-, Pn-status, and tumor grading, are associated with CD96 expression in the tissue and blood samples of OSCC patients. There was no significant association between the CD96 mRNA expression in the peripheral blood samples and the aforementioned parameters.

Additionally, there was no significant correlation between the histomorphological parameters and the CD96 mRNA and protein expression in the tissue specimens (Table 4).

### 4.6. Correlations between CD96 mRNA and Protein Expression, Macrophage and Checkpoint Markers

**Figure 5:**
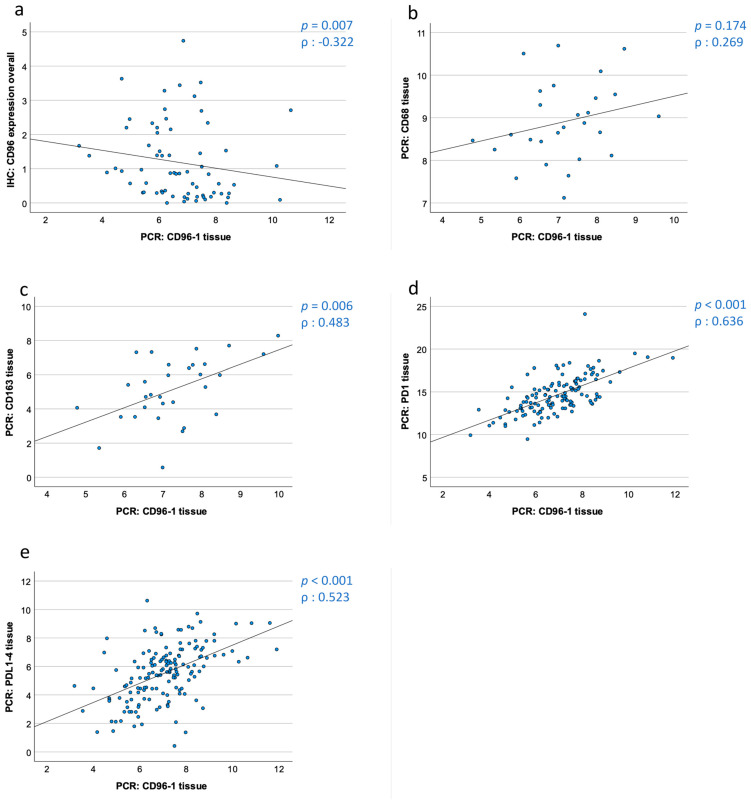
Correlation of CD96 mRNA and protein expression and correlation of CD96 expression with macrophage markers and the PD-L1/PD1 immune checkpoint in tissue samples. The scatter diagrams show the correlation between CD96 mRNA and protein expression (**a**) as well as the correlation of ΔCT values of CD96 (transcript variant 1) with CD68, CD163, PD1, and PD-L1 (transcript variant 4) derived by RT-qPCR analysis (**c**–**e**). Spearmen correlation coefficients (ρ) and the *p*-values are indicated. (**a**) Correlation of CD96 protein expression provided by immunohistochemistry and CD96 mRNA expression (transcript variant 1) provided by RT-qPCR. (**b**) Correlation of CD96 mRNA expression (transcript variant 1) and CD68 mRNA expression provided by RT-qPCR. (**c**) Correlation of CD96 mRNA expression (transcript variant 1) and CD163 mRNA expression provided by RT-qPCR. (**d**) Correlation of CD96 mRNA expression (transcript variant 1) and PD1 mRNA expression provided by RT-qPCR. € Correlation of CD96 mRNA expression (transcript variant 1) and PD-L1 (transcript variant 4) mRNA expression provided by RT-qPCR.

Correlations (Spearman-ρ-correlations) were made only when both methods could be applied to the same patients and if the samples were collected at the same time. First, the correlation between CD96 expression in the tissue specimens (OSCC and healthy mucosa) in RT-qPCR and IHC was checked. A strong negative correlation between the CD96 ∆CT values and the CD96 labeling index obtained by IHC was observed. As low ∆CT values indicate a high mRNA expression, this points to a positive correlation between the CD96 mRNA and protein expression in the tissue samples (p_CD96 IHC_ = 0.005, Spearman correlation_CD96 IHC_ = −0.631) (Table 5 and Figure 5a).

Additionally, the correlation of the CD96 expression with the mRNA expression of the macrophage markers CD68 and CD163 was tested. CD163, with 31 correlated patients, correlated strongly in a positive manner and the collective of 27 CD68 samples did not correlate at all (p_CD68_ = 0.269, p_CD163_ = 0.006, ρ_CD68_ = 0.174, ρ_CD163_ = 0.483) (Table 5 and Figure 5b,c).

Moreover, the CD96 mRNA expression was correlated with the immune checkpoint receptor PD1 and its ligand PD-L1. The expression data of PD1 and PD-L1 were already published [9,10]. The 129 samples of PD-1 and 155 of PD-L1 both strongly correlate with the CD96 tissue expression (p_PD1_ < 0.001, p_PD-L1_ <0.001ρ_PD1_ = 0.636, ρ_PD-L1_ = 0.523) (Table 5 and Figure 5d,e).

## 5. Discussion

### 5.1. CD96 Expression in Tumor Tissue

Murine CD96 was initially identified as an inhibitory immune checkpoint receptor inhibiting the function of T cells and NK cells [19]. CD96 deficient animals had a better control of tumor growth compared to wild-type mice [21]. In humans, it is not yet clear if CD96 can also play an immune-activating role in certain circumstances [19,24].

The current study showed a significantly increased expression of CD96 in the OSCC tumor samples compared to the healthy oral mucosa. This was evident on the mRNA and protein level. In addition, a significant positive correlation between CD96 mRNA and protein expression was proven. The Chi-square test revealed that individual tissue samples can be identified as OSCCs or healthy mucosa depending on their CD96 expression. In contrast, there was no significant association between CD96 expression in the tissue samples and the histomorphological parameters of the OSCC patients.

T cells and NK cells are the main sources of CD96 expression [15]. CD96 is believed to mainly be an inhibitory receptor and CD96 signaling is associated with immunosuppression. Regarding NK cells, especially, exhausted and functionally inactive NK cells are known to have enriched CD96 expression [20]. In addition, CD96-expressing T cells are considered to be functionally exhausted [2]. Since we detected the increased expression of CD96 in the OSCC tissue, this could indicate increased T cell and NK cell exhaustion which is associated with immune inhibitory signaling in the OSCCs compared to the healthy oral mucosa.

Intratumoral NK cells in hepatocellular carcinomas showed a higher CD96 expression compared to the peritumoral tissue [25]. In prostate cancer tissue, high CD96 gene expression was significantly associated with disease recurrence [26]. Human CD96^+^ NK cells in hepatocellular carcinomas were shown to be exhausted with a reduced expression of the immune stimulatory cytokines interferon gamma (IFN-γ) and tumor necrosis factor alpha (TNF-α) and increased expression of the immunosuppressive IL10 and transforming growth factor beta 1 (TGF-β1) [20]. In addition, the CD96^+^ NK cells showed a reduced expression of the killing effector proteins perforin and granzyme B [20]. These results underline the immunosuppressive properties of CD96-expressing cells.

In addition to immune cells, a recent study revealed CD96 expression in human breast cancer cells. High CD96 expression in the tissue samples was associated with poor prognosis [27]. The CD96^+^ breast cancer cells revealed cancer stem cell characteristics and showed increased resistance to chemotherapy [27]. Blocking of CD96 in an immunocompromised xenotransplant mouse model of human breast cancer cells showed reduced tumor growth and increased apoptosis [27]. This is interesting as immune cells play no role in this in vivo model. These data suggest that blocking CD96 may have direct anti-tumor effects independent of the immune system [27]. In the immunohistochemical analysis of the current study, there was a clear dominance of CD96 expression in tumor-infiltrating cells. However, there was also some CD96 staining in epithelial tumor cells as well as epithelial cells in the healthy controls. This indicates that the direct tumor cell-bound expression of CD96 might also be relevant in oral cancer. In the described mouse model, the expression of the CD96 ligand CD155 was also proven on breast cancer cells [27]. This indicates that CD96 signaling can be initiated by tumor cells and that CD155–CD96 interaction contributes to chemoresistance in breast cancer cells independent of immune cells [27]. Blocking of CD96 signaling could, therefore, also have direct immune-independent effects on tumor cells.

CD96 is a relevant player in the CD155/TIGIT/CD96-signaling pathway. Signaling in this pathway might not only be directed from APCs or tumor cells toward CD96-expressing T cells and NK cells but there might also be a tumor cell-derived modification of APC-dependent cytokine production toward immunosuppression via T and NK cells as interim players [28]. This indicates that CD96 signaling is highly complex and not yet fully understood.

Correlation analysis showed a strong positive association between the CD96 and the checkpoint receptor PD1. This is conclusive as it is known that exhausted T cells express high levels of PD1 and CD96 [21]. Tissue samples with increased CD96 and PD1 expression might have an increased level of T cell exhaustion which could contribute to tumor progression.

In addition, a significant positive correlation between PD-L1 and CD96 was found. This indicates that a high expression of the immunosuppressive checkpoint ligand PD-L1, which is mainly found on APCs and tumor cells, is associated with an increased CD96 expression on local T and NK cell populations.

CD8^+^ and CD96^+^ T cells in cervical cancer patients not responding to anti-PD1 ICI treatment often showed a co-expression of CD96 and PD1 [21]. These data motivate to further evaluate a combined checkpoint inhibition of PD1 and CD96. In gastric cancer, high CD96 infiltration was associated with an inferior prognosis [29]. However, gene expression database analysis showed a superior responsiveness of CD96^high^ patients to the anti-PD1 therapy [29]. This result is replicable because the current analysis showed a positive correlation between CD96, PD1, and PD-L1 expression.

Although there was no significant association between CD96 expression and the generic macrophage marker CD68, there was a significant positive correlation between CD96 and the M2 macrophage marker CD163 in the current analysis. M2-polarized macrophages are known to act in an immunosuppressive way and promote tumor growth [30]. In OSCCs, they are associated with metastases and inferior survival [30,31]. In addition, an association with M2-polarized macrophages with PD-L1 expression was already shown [32]. These data indicate that CD96 is associated with other immunosuppressive parameters in the OSCC microenvironment and could contribute to tumor progression.

### 5.2. Potential Therapeutic Use of CD96 Inhibition

CD96 knockout mice showed hyper-reactive NK cells upon immune activation, indicating the inhibitory function of CD96 [33]. In addition, the blocking of CD96 together with PD1 was shown to be efficient against lung metastases in a tumor mouse model [33].

A recent study showed that the deletion of CD96 in human T cells is associated with increased leukemia cell-killing activity in vitro [34]. T cells with a chimeric receptor displaying the extracellular domain of HER2 and the intracellular domain of CD96 showed decreased killing activity against HER2-positive tumor cells compared to T cells lacking the intracellular CD96 domain in vitro and in vivo [34]. As the role of CD96 in human cells is not yet fully understood, these data indicate that immune inhibitory signaling dominates CD96 function in human T cells as well.

Preclinical studies revealed an increased efficiency of immunotherapy if CD96 inhibition was combined with anti-PD1, anti-CTLA4, or anti-TIGIT therapy [35]. Blocking of CD96 showed to increase the activity of cytotoxic CD8^+^ T cells [35].

In lung cancer, prostate cancer, and melanoma mouse models, CD96 inhibition was shown to inhibit tumor metastases [33]. The efficiency of anti-CD96 immunotherapy could be increased in combination with anti-PD1 or anti-CTLA4 [33]. It was shown that the anti-metastatic activity of CD96 blockage was dependent on NK cells [33].

In vitro analyses revealed that tumor-infiltrating lymphocytes obtained from HPV^+^ HNSCC cultivated with anti-CD3 and anti-CD96 antibodies had an increased proliferation compared to a culture with anti-CD3 antibodies [15]. This indicates a potential therapeutic role of CD96 inhibition in HNSCC [15]. The increased expression of CD96 in OSCCs shown by the current study also motivates the investigation of anti-CD96 therapy in oral cancer. This could reverse NK cell exhaustion [20] and increase the activation of T cells [34].

### 5.3. CD96 Expression in Peripheral Blood

In contrast to the tissue samples, there was a significantly decreased CD96 mRNA expression in the peripheral blood of the OSCC patients compared to the healthy control persons.

This assumption is supported by several studies. In pancreatic cancer patients, a decreased proportion of CD96^+^ NK cells was found in the peripheral blood compared with the healthy controls [16,25]. A possible reason for the decreased CD96 expression in the peripheral blood of the OSCC patients could be a shift of CD96-expressing cells from the periphery to the tumor site.

In addition, the Chi-square test revealed that individual blood samples can be identified as originating from an OSCC patient or a healthy control person depending on their CD96 expression. As the sensitivity and specificity of this test are relatively low, it is not suitable as a single diagnostic blood test for OSCC. However, CD96 expression in the peripheral blood could contribute to a multi-marker liquid biopsy for OSCC diagnosis and monitoring and should, therefore, be further investigated.

CD96 expression in the peripheral blood was not associated with histomorphological parameters. In contrast to this, a high PD-L1 expression in the peripheral blood was significantly associated with lymph node metastases and inferior overall survival in OSCCs [9,11]. Similar results were found in surgically treated esophageal cancer, where the preoperative serum level of PD-L1 was associated with inferior survival [36].

## 6. Conclusions

The current study shows increased CD96 mRNA and protein expression in oral cancer tissue compared to the healthy oral mucosa. This could be a reason for impaired T-cell and NK-cell function in OSCCs, leading to immunosuppression. Hence, CD96 seems to be a relevant immune checkpoint and may be a suitable target for ICI therapy. In addition, CD96 expression in OSCC tissue was positively associated with PD1 and PD-L1 which are important ICPs in oral cancer and are used successfully in immune therapy. Hence, a combination of the ICI could increase the effectiveness of the treatment in OSCCs as was shown for other solid tumors.

CD96 expression in the peripheral blood of OSCC patients was significantly decreased and the downregulation was significantly associated with the diagnosis of malignancy. Therefore, the value of CD96 expression for a multi-marker liquid biopsy for oral cancer should be evaluated. Additional research is needed to further elucidate the exact function of the CD96 immune checkpoint pathway in human cancer.

## Figures and Tables

**Table 1:** Demographic characteristics of controls and OSCC patients. Demographic characteristics of healthy volunteers (control group) and OSCC patients (patient group) for CD96 analysis. For the patient group, parameters for, T-status, N-status, L-status, Pn-status, and grading are shown. For both OSCC and controls, group characteristics of gender, number of cases, and age are shown. Exact data for RT-qPCR (a) and immunohistochemistry (b) analyses are given.

(a)		Patients (OSCC)		Healthy Controls	
		n	% of cases	n	% of cases
**Number of cases PCR**	183	98	54	85	46
**Gender**	Male	65	66.3	51	60
	Female	33	33.7	34	40
**Mean age ± SD**		62.66 ± 11.7 years		49.7 ± 19.7 years	
**Range of age**		31–93 years		18–88 years	
			% of cases		
**T-Status**	T1	28	28.6		
	T2	33	33.7		
	T3	14	14.3		
	T4	18	18.4		
	Unknown	5	5		
**N-Status**	N0	53	54.1		
	N1	13	13.3		
	N2a	13	13.3		
	N2b	15	15.3		
	N2c	3	3		
	Unknown	1	1		
**L-Status**	L0	70	71.4		
	L1	18	18.4		
	Unknown	10	10.2		
**Pn-Status**	Pn0	59	60.2		
	Pn1	28	28.6		
	Unknown	11	11.2		
**Grading**	G1	9	9.2		
	G2	46	47		
	G3	37	37.7		
	Unknown	6	6.1		
**(b)**		**Patients (OSCC)**		**Healthy Controls**	
		n	% of cases	n	% of cases
**Number of cases IHC**	141	90	64	51	36
**Gender**	Male	62	69	38	74.5
	Female	28	31	13	25.5
**Mean age ± SD**		63.4 ± 12.4 years		48.7 ± 19.1 years	
**Range of age**		33–89 years		18–87 years	
			% of cases		
**T-Status**	T1	17	18.9		
	T2	34	37.8		
	T3	14	15.5		
	T4	23	25.6		
	Unknown	2	2.2		
**N-Status**	N0	50	55.6		
	N1	13	14.5		
	N2a	10	11.1		
	N2b	12	13.3		
	N2c	2	2.2		
	Unknown	3	3.3		
**L-Status**	L0	60	66.7		
	L1	16	17.8		
	Unknown	14	15.5		
**Pn-Status**	Pn0	52	57.8		
	Pn1	26	28.9		
	Unknown	12	13.3		
**Grading**	G1	4	4.5		
	G2	47	52.2		
	G3	30	33.3		
	Unknown	9	10		

**Table 2:** Real-time qPCR primer. The table shows selected primers for RT-qPCR mRNA expression analysis of PD1, CD96_1, CD96_3, PD-L1_4, GAPDH, CD68, and CD163.

Primer	Sequence (5′ to 3′)	Primer (bp)	Amplicon (bp)	Annealing Temp. (°C)	Accession
CD96_1 s	ACCTCCAGTGGGACAGATACC	21	91	60	NM_198196.3 *
CD96_1 as	GAAGTGTTGAGCCTGCACCT	20	–	–	
CD96_3 s	GCATGGTCGGTGGAGGATAA	20	130	60	NM_001318889 **
CD96_3 as	GGACTGGAGAGAGGTGGAGT	20	–	–	
PD1 s	AAACCCTGGTGGTTGGTGTC	20	105	–	NM_005018.2
PD1 as	CTCCTATTGTCCCTCGTGCG	20	–	–	
PD-L1 s	AGCTATGGTGGTGCCGACTA	20	152	60	NM_014143.3 ^§^
PD-L1 s	CAGATGACTTCGGCCTTGGG	20	–	–	NM_001314029.1
CD68 s	TGGGTGGGATCATCTCCAGT	20	100	60	NM_001040059.1 ^$^ NM_001251.2 ^$^
CD68 as	TAGGCTGTCTGCACCAGTTG	20	–	60	
CD163 s	CTTGGGGTTGTTCTGTTGGC	20	92	60	NM_004244 ^++^
CD163 as	CCTCTTGAGGAAACTGCAAGC	21	–	60	
GAPDH s	GACCCCTTCATTGACCTCAACTA	23	102	60	NM_002046.5
GAPDH as	GAATTTGCCATGGGTGGAAT	20	–	–	

* Amplification of transcript variant 1 of CD96 that encodes the longer isoform (detection of all isoforms except of transcript variant 3); ** amplification of transcript variant 3 of CD96 that encodes a shorter isoform with a distinct C-terminus compared to variant 1; ^++^ transcript variant 1; amplicon generated from various isoforms (1–3, 5); ^§^ amplification of transcript variant 1 and 4simultaneously; amplicon named PD-L1; ^$^ CD68 accession number of the year 2018; new record available.

**Table 3:** CD96 expression in tissue and peripheral blood of healthy controls and OSCC patients. This table compares CD96 expression in peripheral blood and tissue samples, analyzed with RT-qPCR and immunohistochemistry (IHC), between the control and OSCC groups. The mean ΔCT value (mean) for RT-qPCR and the labelling index (mean) for IHC, standard deviation (SD), and the p-value provided by the Mann–Whitney U test are shown. Higher labelling indices (LI) indicate higher CD96 expression, whereas a high ∆CT value indicates a lower CD96 expression. Regarding the CD96 expression, the area under the curve (AUC), fold change (FC), and cut-off point (COP) values are given. The controls and patients were labelled as positive and negative, based on their mean ΔCT value or labelling index regarding the calculated COP value. Furthermore, the percentage (% pos. cases) of positive-tested cases in the control and the patient group is shown. n.d.: not determined.

	n	Mean	SD	*p*-Value	AUC	FC	COP	No. of Cases	+	−	% Pos. Cases	*p*-Value χ^2^ Test	Sensitivity	Specificity	Pos. Predictive Value	Neg. Predictive Value
**RT-qPCR** **CD96_1 blood**		∆CT		**0.014**	0.609	0.84	4.29	172				**0.012**	0.529	0.282	0.43	0.369
controls	85	3.99	0.70					85	61	24	71.8					
patients	87	4.24	0.73					87	46	41	52.9					
**RT-qPCR** **CD96_3 blood**		∆CT		0.095	n.d.	n.d.	n.d.	n.d.	n.d.	n.d.	n.d.	n.d.	n.d.	n.d.	n.d.	n.d.
controls	85	3.40	0.74													
patients	87	3.59	0.74													
**RT-qPCR** **CD96_1** **tissue**		∆CT		**0.012**	0.615	1.28	6.52	166				**0.003**	0.469	0.765	0.742	0.5
controls	68	7.20	1.48					68	16	52	23.5					
patients	98	6.84	1.48					98	46	52	46.9					
**RT-qPCR** **CD96_3** **tissue**		∆CT		**<0.001**	0.652	1.52	9.27	164					0.656	0.618	0.708	0.56
controls	68	9.38	1.46					68	26	42	38.2	**<0.001**				
patients	96	8.78	1.55					96	63	33	65.6					
**IHC** **CD96** **tissue overall**		LI		**0.003**	0.650	1.53	0.905	141				**0.005**	0.567	0.686	0.761	0.473
controls	51	0.91	1.15					51	16	35	31.4					
patients	90	1.39	1.15					90	51	39	56.7					
**IHC** **CD96** **tissue** **epithelium**		LI		**0.011**	0.629	1.28	0.325	141				**0.003**	0.733	0.529	0.733	0.529
controls	51	0.85	1.41					51	24	27	47.1					
patients	90	1.09	1.00					90	66	24	73.3					
**IHC** **CD96** **tissue** **stroma**		LI		**0.008**	0.638	1.39	0.715	135				**0.007**	0.671	0.58	0.731	0.509
controls	50	1.52	3.72					50	21	29	42.0					
patients	85	2.11	2.94					85	57	28	67.1					

**Table 4:** CD96 expression in tissue of OSCC patients related to histomorphological parameters. (T-, N-, L-, Pn-status, grading). The association with the CD96 expression in tissue specimens of the patient group to histomorphological parameters (T-, N-, L-, Pn-status, grading) is shown in Table 4. The samples were analyzed via RT-qPCR and IHC. Displayed are the mean ΔCT value (mean) for RT-qPCR and the labelling index (mean) for IHC, the standard deviation (SD). The p-value was determined by the Mann–Whitney U test. Higher labelling indices (LI) indicate a higher CD96 expression, whereas higher ∆CT values indicate a lower CD96 expression.

		N	Mean	SD	*p*-Value
	**PCR** **CD96_1 tissue**	93			0.258
**T-status**	T1-T2	61	6.9	1.41	
	T3-T4	32	6.60	1.38	
	**IHC** **CD96**	88			0.264
	T1-T2	51	1.52	1.23	
	T3-T4	37	1.22	1.03	
	**PCR** **CD96_1 tissue**	94			0.417
**N-status**	N0	53	6.91	1.46	
	N+	41	6.64	1.31	
	**IHC** **CD96**	87			0.315
	N0	50	1.52	1.23	
	N+	37	1.26	1.06	
	**PCR** **CD96_1 tissue**	88			0.975
**L-status**	L0	70	6.77	1.44	
	L1	18	6.80	1.47	
	**IHC** **CD96**	80			0.336
	L0	64	1.50	1.19	
	L1	16	1.25	1.11	
	**PCR** **CD96_1 tissue**	87			0.322
**Pn-status**	Pn0	59	6.68	1.28	
	Pn1	28	7.18	1.60	
	**IHC** **CD96**	78			0.090
	Pn0	52	1.61	1.24	
	Pn1	26	1.12	1.02	
	**PCR** **CD96_1 tissue**	92			0.606
**grading**	G1	9	6.56	1.17	
	G2	46	6.65	1.38	
	G3	37	7.01	1.51	
	**IHC** **CD96**	81			0.445
	G1	4	2.27	1.76	
	G2	47	1.42	1.16	
	G3	30	1.35	1.13	

**Table 5:** Correlation of CD96_1 tissue with CD96 IHC, CD68, CD163, PD-1, and PDL1_4 expression in tissue specimens.

		CD96_1 PCR Tissue
CD96 IHC tissue	Spearman correlation	−0.631
	*p*-value	0.005 *
	n	18
CD68 PCR tissue	Spearman correlation	0.174
	*p*-value	0.269
	n	27
CD163 PCR tissue	Spearman correlation	0.483
	*p*-value	0.006 *
	n	31
PD-1 PCR tissue	Spearman correlation	0.636
	*p*-value	<0.001 *
	n	129
PDL1_4 PCR tissue	Spearman correlation	0.523
	*p*-value	<0.001 *
	n	155

Correlation of CD96 mRNA with protein expression and correlation of CD96 expression with CD68, CD163, PD-1, and PD-L1. Table 5 shows the correlation of CD96_1 mRNA expression (derived by RT-qPCR) in tissue specimens with CD96 protein expression (derived by IHC), CD68 (mRNA), CD163 (mRNA), CD163 (mRNA), PD-1 (mRNA), and PDL1_4 (mRNA). Values represent the number of cases (n), Spearman correlation coefficient, and *p*-value. Significant correlations are marked with an *.

## Data Availability

Primary data are available upon request form the authors.
